# Bilateral Carpal Tunnel Syndrome Induced by Nivolumab Immunotherapy

**DOI:** 10.7759/cureus.95866

**Published:** 2025-10-31

**Authors:** Amjed A Alhilfi, Serena Hilman

**Affiliations:** 1 Oncology, University Hospitals Bristol and Weston NHS Foundation Trust, Bristol, GBR

**Keywords:** carpal tunnel syndrome, immune checkpoint inhibitors, immune-related adverse events, kidney cancer, nivolumab, peripheral neuropathy

## Abstract

Checkpoint inhibitors have been the cornerstone in the treatment of many advanced cancers. Despite their efficacy, these therapies can lead to a wide range of immune-related adverse events, some of which may cause neurological toxicity and present significant clinical challenges. We describe the case of a 78-year-old man who developed bilateral carpal tunnel syndrome while receiving nivolumab for metastatic collecting duct carcinoma of the kidney. His symptoms showed marked improvement following corticosteroid therapy and the discontinuation of immunotherapy. This case underscores the critical need for the prompt recognition and collaborative management of neurological immune-related adverse events to optimize patients' outcomes.

## Introduction

Neurological complications from checkpoint inhibitor therapy are relatively rare, affecting approximately 0.3-1% of patients [1-3]. These issues typically arise within 2-12 weeks after treatment begins [4]. Reported neurological syndromes include inflammatory myopathies, myasthenia gravis, acute and chronic demyelinating polyradiculopathies such as Guillain-Barré syndrome, vasculitic neuropathies, isolated cranial nerve palsies, aseptic meningitis, autoimmune encephalitis, and multiple sclerosis [4].

Carpal tunnel syndrome (CTS) is the most common type of entrapment neuropathy, affecting 3-6% of the general population [2]. Symptoms typically include pain, tingling, and numbness in areas served by the median nerve, sometimes radiating to the forearm, upper arm, or shoulder. Patients may also report decreased grip strength or difficulty performing fine motor tasks.

CTS is frequently associated with repetitive hand or wrist motions, often related to occupational activities. Common at-risk professions include assembly line workers, cashiers, administrative professionals who spend long hours typing, and those in manufacturing roles requiring repeated wrist movements [2,3]. The patient in this case worked as a builder, an occupation also associated with repetitive wrist use. Despite its prevalence in the general population, a link between checkpoint inhibitor use and the development of CTS is exceptionally rare and not well understood.

While similar cases have been reported, this case highlights the diagnostic challenges in distinguishing immune-mediated neuropathy from age- or occupation-related CTS. It also underscores the importance of considering immune-related mechanisms when bilateral, rapidly progressive symptoms develop during checkpoint inhibitor therapy.

## Case presentation

A 78-year-old gentleman with metastatic collecting duct carcinoma of the kidney initially underwent a left radical nephrectomy in November 2021. In July 2023, surveillance CT of the chest, abdomen, and pelvis revealed lung metastases and a new 15 mm cavitating lesion in the upper lobe with possible lymphangitis carcinomatosis. This CT scan was also discussed in the urology multidisciplinary team's complex cases, and the outcome was to consider first-line carboplatin and gemcitabine chemotherapy, which achieved a period of stable disease until January 2024, when a CT of the chest, abdomen, and pelvis showed the progression of lung metastases. At that time, he was commenced on four-weekly nivolumab immunotherapy. From an oncology standpoint, the disease remained stable throughout 2024 and was well tolerated. However, by early 2025, after receiving 11 cycles of nivolumab (approximately eight months after treatment initiation), he started experiencing increasing discomfort in both hands. His symptoms included numbness, nocturnal pain, and swelling, particularly affecting the wrists and fingers. These symptoms progressively began to interfere with his daily activities and hand function. He noted increasing difficulty with gripping objects, and his sleep quality was significantly impacted, as he frequently woke during the night due to hand discomfort.

On clinical examination, the patient was noted to have swelling in both hands, with the left hand being more affected. Phalen's test was carried out as part of the neurological evaluation. This involves asking the patient to bring the backs of the hands together with the wrists fully flexed and to hold the position for about half a minute. The patient reported tingling and discomfort radiating to the fingers supplied by the median nerve bilaterally, indicating likely compression at the carpal tunnel. These findings were suggestive of CTS.

In addition to this, the patient described pain extending along the course of the ulnar nerve, particularly in the ring fingers on both sides. This raised the possibility of concurrent involvement of the ulnar nerve. There was no evidence of joint inflammation in the wrist, metacarpophalangeal, and interphalangeal joints, which made an inflammatory joint condition such as rheumatoid arthritis seem less likely.

Given the combination of the symptoms, we sought input from the rheumatology team. Their advice was to proceed with nerve conduction studies to better understand the pattern and severity of the nerve involvement. In the meantime, they recommended a specialized wrist splint supplied by the occupational therapy team to help with the symptoms.

Initial management included pregabalin and codeine, which offered only partial relief. He was seen by the general practitioner (GP), who arranged autoimmune screening, including rheumatoid factor and antinuclear antibodies, all of which returned negative. Interestingly, while hospitalized in March 2025 for a chronic obstructive pulmonary disease (COPD) exacerbation, he received a course of prednisolone, during which he noted a temporary improvement in his hand symptoms. This response raised the suspicion of an underlying immune-mediated inflammatory process.

Other causes such as diabetes mellitus, thyroid dysfunction, and B12 deficiency were excluded through appropriate blood tests, all of which were normal. Nerve conduction studies carried out on March 6, 2025, confirmed severe bilateral median neuropathies, most pronounced on the right alongside features of a more generalized axonal peripheral neuropathy. There was also a mild involvement of the right ulnar nerve (Table 1 and Table 2).

**Table 1:** Sensory nerve conduction study findings NR: not recorded; Pk-p: peak-to-peak; R: right; L: left

Nerve/site	Rec. site	Latency (ms)	Peak amplitude (µV)	Pk-p amplitude (µV)	Distance (cm)	Velocity (m/s)
R median: ulnar DII-V	Wrist	-	-	-	-	-
Index	Wrist	NR	NR	NR	-	-
Middle	Wrist	NR	NR	NR	-	-
Little	Wrist	2.29	0.97	2.5	11	48.0
R median: ulnar palm to wrist	Wrist	-	-	-	-	-
Median palm	Wrist	NR	NR	NR	8	NR
Ulnar palm	Wrist	1.48	3.5	6.9	8	54.1
R radial	Forearm	2.46	4.6	9.0	13.5	54.9
L median: ulnar DII-V	Wrist	-	-	-	-	-
Index	Wrist	NR	NR	NR	-	-
Middle	Wrist	NR	NR	NR	-	-
Little	Wrist	2.42	1.9	4.1	10.5	43.4
L median: ulnar palm to wrist	Wrist	-	-	-	-	-
Median palm	Wrist	NR	NR	NR	8	NR
Ulnar palm	Wrist	1.67	3.9	7.9	8	48.0
L radial	Forearm	2.44	5.2	8.0	14	57.4

**Table 2:** Motor nerve conduction study findings ADM: abductor digiti minimi; APB: abductor pollicis brevis; FDI: first dorsal interosseous; R: right; L: left

Nerve/site	Site	Latency (ms)	Amplitude (mV)	Distance (cm)	Velocity (m/s)
R median: APB	Wrist	7.02	3.3	24	45.2
R median: APB	Elbow	12.33	2.6	14	45.7
R median: APB	Axilla	15.40	2.9	-	-
R ulnar: ADM	Wrist	1.98	9.0	26	58.6
R ulnar: ADM	Below elbow	6.42	8.8	9.5	39.0
R ulnar: ADM	Above elbow	8.85	8.8	10	57.8
R ulnar: ADM	Axilla	10.58	8.7	-	-
R ulnar: FDI	Wrist	2.96	4.8	26	51.8
R ulnar: FDI	Below elbow	7.98	4.4	9.5	44.7
R ulnar: FDI	Above elbow	10.10	4.0	10	52.2
R ulnar: FDI	Axilla	12.02	3.6	-	-
L median: APB	Wrist	6.02	5.9	24	43.6
L median: APB	Elbow	11.52	4.8	12	66.2
L median: APB	Axilla	13.33	4.8	-	-
L ulnar: ADM	Wrist	2.46	7.3	25.5	55.4
L ulnar: ADM	Below elbow	7.06	7.3	9.5	51.8
L ulnar: ADM	Above elbow	8.90	7.3	-	-

Considering the progression of symptoms and the timing in relation to immunotherapy, nivolumab was suspended after the 13th cycle. On March 27, 2025, the patient was started on oral prednisolone at 60 mg daily, with the planned taper of 10 mg per week. By the follow-up review on April 24, 2025, he reported a marked improvement in his symptoms. The swelling had resolved, nocturnal pain had subsided, and hand function was noticeably better. On examination, there were no lingering neurological deficits. 

After the discussion of the risks and benefits, it has been decided to permanently discontinue nivolumab, prioritizing quality of life and symptom control. A CT scan from March 2025 showed stable disease, and he remained under clinical surveillance. The repeat scan in June and September 2025 also confirmed disease stability.

Approximately one week after completing the steroid taper, symptoms recurred. Prednisolone 10 mg daily was restarted and then escalated to 30 mg before tapering to 15 mg daily. Persistent symptoms prompted a rheumatology referral, who advised orthopedic assessment for possible surgical decompression. Hence, prednisolone was reintroduced at 10 mg once daily for 10 days, followed by 5 mg daily with a review scheduled after three weeks.

At the follow-up three weeks later, the patient explained that the low dose had not really helped. The decision was made to increase prednisolone to 30 mg daily for a week and then taper down to 20 mg once daily for two weeks, followed by 15 mg once daily.

Given the persistent symptoms, the rheumatology team was consulted. Their recommendation was that an orthopedic opinion should be sought to look at the possibility of surgical treatment for his CTS, and a referral was arranged.

## Discussion

Immune checkpoint inhibitors such as nivolumab have significantly changed the treatment landscape of advanced cancers but are associated with a wide range of immune-related adverse effects. Although less common than other toxicities, neurological adverse effects can be debilitating and challenging to manage, often having a notable impact on patient wellbeing [1,5].

The patient developed bilateral CTS confirmed on nerve conduction studies following the initiation of nivolumab. The patient's symptoms worsened during immunotherapy but improved significantly with corticosteroid, aligning with other reports of immune-related neuropathies [1,6].

The pathogenesis of checkpoint inhibitor-associated neuropathies is incompletely understood. Proposed mechanisms include immune-mediated neuritis of the median nerve, perineural inflammation, or systemic autoimmune activation resulting in axonal injury. The bilateral onset, absence of occupational exacerbation, and relapse after steroid taper support an immune-mediated contribution rather than purely mechanical compression. However, the patient's age and manual labor history remain relevant risk factors, making this a probable rather than definitive association.

In the general population, CTS is found to have a notable predilection for women, reported at a sex ratio approaching 10:1 [7].

Anatomically, CTS results from the compression of the median nerve as it traverses the carpal tunnel, a confined space bordered anteriorly by the flexor retinaculum and posteriorly by the carpal bones. Within this space lie the flexor tendons of the fingers, the flexor pollicis longus, and the median nerve, a key neural structure originating from the brachial plexus. This nerve is responsible for both sensory and motor innervation: it supplies sensation to the palmar aspects of the thumb, index finger, middle finger, and radial half of the ring finger, as well as the distal dorsal surfaces of these digits. Functionally, it enables thumb opposition and contributes to thumb abduction and flexion via the thenar muscles, in addition to innervating the lateral lumbricals that facilitate metacarpophalangeal flexion and interphalangeal extension [7].

Clinical manifestations of CTS are variable but frequently include paresthesia, numbness, and discomfort localized to the median nerve distribution. In more advanced or prolonged cases, patients may also exhibit motor impairment, particularly involving the thumb and index finger. These symptoms are consistent with both idiopathic and immune checkpoint inhibitor-induced presentations.

Nerve conduction studies in our case revealed severe bilateral median neuropathy and signs of broader axonal damage which are overall consistent with immune-related peripheral nerve injury. The presence of mild ulnar involvement further supported the possibility of a generalized process rather than isolated mechanical entrapment. Such cases underscore the need for clinicians to distinguish between typical degenerative CTS and inflammatory presentations secondary to immune checkpoint inhibitors.

In our patient, the electrophysiological findings demonstrated markedly prolonged distal latencies and reduced sensory amplitudes in the median nerves, with the partial involvement of the ulnar nerves bilaterally. This was accompanied by reduced conduction velocities and decreased compound muscle action potential amplitudes in motor studies. Such findings extend beyond localized mechanical compression at the carpal tunnel and are more suggestive of a diffuse inflammatory or immune-mediated axonal neuropathy. The coexistence of ulnar nerve abnormalities, typically spared in idiopathic CTS, further supports a generalized process rather than isolated entrapment. When considered alongside the temporal relationship between nivolumab administration, corticosteroid responsiveness, and symptom fluctuations, these electrophysiological patterns reinforce the hypothesis of an immune-related neuropathy rather than a purely degenerative or occupational etiology.

The successful response to corticosteroids in this patient reflects current guidance for the management of moderate to severe immune-related adverse events (irAEs), which recommends immunosuppression alongside the interruption of immunotherapy [8]. In this situation, the patient prioritized symptom control and opted for the permanent discontinuation of nivolumab. However, his symptoms recurred following steroid withdrawal, necessitating reintroduction and subsequent dose escalation. This relapsing course is consistent with other immune-related neuropathies and underlines the need for cautious tapering, close follow-up, and multidisciplinary input.

Several published case reports have described similar instances of CTS linked to immune checkpoint inhibitor therapy. For example, Lechevalier and colleagues outlined 11 patients who developed bilateral CTS during treatment, with some of them undergoing surgery [9]. Yakobson et al. discussed four patients with immune checkpoint inhibitor-induced CTS that resolved following corticosteroid and immunosuppressive therapy [10]. Another series by Shalata et al. described three patients who improved with immune-modulating treatments [11]. Additionally, Eisenbud and coworkers documented two patients without any prior CTS history who developed bilateral symptoms while being on immune checkpoint inhibitors [12]. In a separate report, Gorzelak-Magiera et al. highlighted a patient treated with pembrolizumab who developed symptoms suggestive of CTS after prolonged therapy [13].

These reports collectively suggest that immune checkpoint inhibitor-induced CTS is often bilateral and may present with more severe and acute symptoms compared to idiopathic CTS. In contrast, idiopathic CTS usually has a gradual onset, frequently begins unilaterally, and is more likely to initially affect the dominant hand [9-12]. This distinction is clinically important, as bilateral and rapid-onset CTS in patients receiving immune checkpoint inhibitors may be a marker of an immune-related adverse event rather than a typical mechanical neuropathy [14-17].

## Conclusions

This case illustrates a probable association between nivolumab therapy and bilateral CTS, a rare but clinically significant immune-related complication. Clinicians should maintain vigilance for neurological irAEs, especially when typical conditions such as CTS present bilaterally or atypically during immunotherapy. Early recognition, corticosteroid intervention, and multidisciplinary coordination are key to optimizing outcomes while balancing cancer control and quality of life.

## References

[1] Dubey D, David WS, Amato AA (2019). Varied phenotypes and management of immune checkpoint inhibitor-associated neuropathies. Neurology.

[2] LeBlanc KE, Cestia W (2011). Carpal tunnel syndrome. Am Fam Physician.

[3] Palmer KT (2011). Carpal tunnel syndrome: the role of occupational factors. Best Pract Res Clin Rheumatol.

[4] Dalakas MC (2018). Neurological complications of immune checkpoint inhibitors: what happens when you 'take the brakes off' the immune system. Ther Adv Neurol Disord.

[5] Spain L, Walls G, Julve M (2017). Neurotoxicity from immune-checkpoint inhibition in the treatment of melanoma: a single centre experience and review of the literature. Ann Oncol.

[6] Cuzzubbo S, Javeri F, Tissier M (2017). Neurological adverse events associated with immune checkpoint inhibitors: review of the literature. Eur J Cancer.

[7] Genova A, Dix O, Saefan A, Thakur M, Hassan A (2020). Carpal tunnel syndrome: a review of literature. Cureus.

[8] Puzanov I, Diab A, Abdallah K (2017). Managing toxicities associated with immune checkpoint inhibitors: consensus recommendations from the Society for Immunotherapy of Cancer (SITC) Toxicity Management Working Group. J Immunother Cancer.

[9] Lechevalier D, Denis D, Le Corre Y (2021). Carpal tunnel syndrome: a new adverse effect of immune checkpoint inhibitors, 11 cases. J Immunother.

[10] Yakobson A, Rouvinov K, Cohen AY (2023). Carpal tunnel syndrome associated with immune checkpoint inhibitors. J Pers Med.

[11] Shalata W, Yakobson A, Massalha I (2020). Carpal tunnel syndrome as an unusual immune checkpoint inhibitor adverse effect: a case series and review of the literature. Int J Inn Res Med Sci.

[12] Eisenbud L, Ejadi S, Mar N (2021). Development of carpal tunnel syndrome in association with checkpoint inhibitors. J Oncol Pharm Pract.

[13] Gorzelak-Magiera AS, Domagała-Haduch M, Gisterek-Grocholska I (2024). Complications of immunotherapy in the course of long-term response in a patient with non-small cell lung cancer. Oncol Clin Pract.

[14] Atroshi I, Gummesson C, Johnsson R, Ornstein E, Ranstam J, Rosén I (1999). Prevalence of carpal tunnel syndrome in a general population. JAMA.

[15] Padua L, Padua R, Aprile I, Pasqualetti P, Tonali P (2001). Multiperspective follow-up of untreated carpal tunnel syndrome: a multicenter study. Neurology.

[16] Dale AM, Harris-Adamson C, Rempel D (2013). Prevalence and incidence of carpal tunnel syndrome in US working populations: pooled analysis of six prospective studies. Scand J Work Environ Health.

[17] You D, Smith AH, Rempel D (2014). Meta-analysis: association between wrist posture and carpal tunnel syndrome among workers. Saf Health Work.

